# Case report: Personalized transcatheter approach to mid-aortic syndrome by *in vitro* simulation on a 3-dimensional printed model

**DOI:** 10.3389/fcvm.2022.1076359

**Published:** 2023-01-10

**Authors:** Luca Giugno, Giovanni Maria Formato, Massimo Chessa, Emiliano Votta, Mario Carminati, Francesco Sturla

**Affiliations:** ^1^Department of Pediatric and Adult Congenital Cardiology, IRCCS Policlinico San Donato, San Donato Milanese, Italy; ^2^3D and Computer Simulation Laboratory, IRCCS Policlinico San Donato, San Donato Milanese, Italy; ^3^Facoltà di Medicina e Chirurgia, Vita Salute San Raffaele University, Milan, Italy; ^4^European Reference Network for Rare and Low Prevalence Complex Diseases of the Heart: ERN GUARD-Heart, Amsterdam, Netherlands; ^5^Department of Electronics, Information and Bioengineering, Politecnico di Milano, Milan, Italy

**Keywords:** stenting, 3D printing, *in vitro* simulation, mid-aortic syndrome, interventional cardiology

## Abstract

An 8-year-old girl, diagnosed with mid-aortic syndrome (MAS) at the age of 2 months and under antihypertensive therapy, presented with severe systemic hypertension (>200/120 mmHg). Computed tomography (CT) examination revealed aortic aneurysm between severe stenoses at pre- and infra-renal segments, and occlusion of principal splanchnic arteries with peripheral collateral revascularization. Based on CT imaging, preoperative three-dimensional (3D) anatomy was reconstructed to assess aortic dimensions and a dedicated *in vitro* planning platform was designed to investigate the feasibility of a stenting procedure under fluoroscopic guidance. The *in vitro* system was designed to incorporate a translucent flexible 3D-printed patient-specific model filled with saline. A covered 8-zig 45-mm-long Cheatham-Platinum (CP) stent and a bare 8-zig, 34-mm-long CP stent were implanted with partial overlap to treat the stenoses (global peak-to-peak pressure gradient > 60 mmHg), excluding the aneurysm and avoiding risk of renal arteries occlusion. Percutaneous procedure was successfully performed with no residual pressure gradient and exactly replicating the strategy tested *in vitro*. Also, as investigated on the 3D-printed model, additional angioplasty was feasible across the frames of the stent to improve bilateral renal flow. Postoperative systemic pressure significantly reduced (130/70 mmHg) as well as dosage of antihypertensive therapy. This is the first report demonstrating the use of a 3D-printed model to effectively plan percutaneous intervention in a complex pediatric MAS case: taking full advantage of the combined use of a patient-specific 3D model and a dedicated *in vitro* platform, feasibility of the stenting procedure was successfully tested during pre-procedural assessment. Hence, use of patient-specific 3D-printed models and *in vitro* dedicated platforms is encouraged to assist pre-procedural planning and personalize treatment, thus enhancing intervention success.

## Introduction

Mid-aortic syndrome (MAS) is a rare disease presenting in pediatric patients with segmental narrowing of the abdominal or distal thoracic aorta and involvement of renal and splanchnic arterial branches, with about 60% of cases with renal involvement exhibiting bilateral renal artery stenosis (1–3). Notably, more than 90% pediatric MAS patients require medical therapy for severe and uncontrolled hypertension (4), which is recognized as major symptom of MAS in children and can lead to renal failure, congestive heart failure, and cerebrovascular accidents (1, 5, 6). However, medical management of refractory hypertension is often insufficient, thus requiring percutaneous, i.e., balloon angioplasty and stenting, or surgical intervention (5).

To date, accepted and standardized guidelines for invasive management of MAS are still lacking (1, 4) and selection of the most appropriate invasive strategy depends on multiple factors such as MAS anatomical complexity, severity of arterial stenosis, patient age and clinical condition (e.g., renal impairment, cardiomyopathy, etc.) and risk of end-organ damage.

Hence, adequate preoperative planning may be crucial to assess the feasibility of invasive procedures with the final aim to increase intervention success and reduce the risk of postoperative complications. We herein present a case of a young girl, presenting with severe MAS and infrequent aortic wall aneurysm, successfully treated planning *in vitro* the percutaneous intervention on a 3-dimensional (3D) printed model.

## Case description

An 8-year-old, 27 Kg weight and 129 cm height (body surface area of 0.99 m^2^) girl presented to our unit due to severe systemic hypertension (>200/120 mmHg), despite antihypertensive therapy. She was diagnosed with idiopathic MAS at the age of 2 months while presenting with heart failure and underwent rescue angioplasty due to severe abdominal aortic coarctation. At the age of 1 year the baby underwent redo angioplasty due to evidence of severe re-coarctation. At follow up, the baby showed severe systemic hypertension, thus requiring multiple antihypertensive therapy. At the age of seven, computed tomography (CT) examination revealed an aortic aneurysm between severe stenoses at pre- and infrarenal segments, and occlusion of principal splanchnic arteries with peripheral collateral revascularization, and sub-occlusion of renal arteries. A speculative hypothesis of iatrogenic aortic aneurysm was made according to the previous clinical history of the baby, the idiopathic etiology of MAS and the specific localization of the aortic aneurysm.

Accordingly, the young girl underwent cardiac catheterization, which confirms the severe stenosis of pre-renal and infrarenal tract of abdominal aorta (gradient peak to peak > 60 mmHg) with evidence of aortic wall aneurysm between the stenosis and bilateral sub-occlusion of renal artery. Priority was given to the bilateral sub-occlusion of renal arteries and renal artery angioplasty with non-compliant coronary balloons was performed to improve renal artery flow. However, despite the significant renal flow recovery, an almost negligible reduction of systemic blood pressure (185/115 mmHg) was noticed.

More recently, a CT scan was performed to monitor the level of stenosis of renal arteries and assess the feasibility of a percutaneous treatment for aortic coarctation.

**Figure 1:**
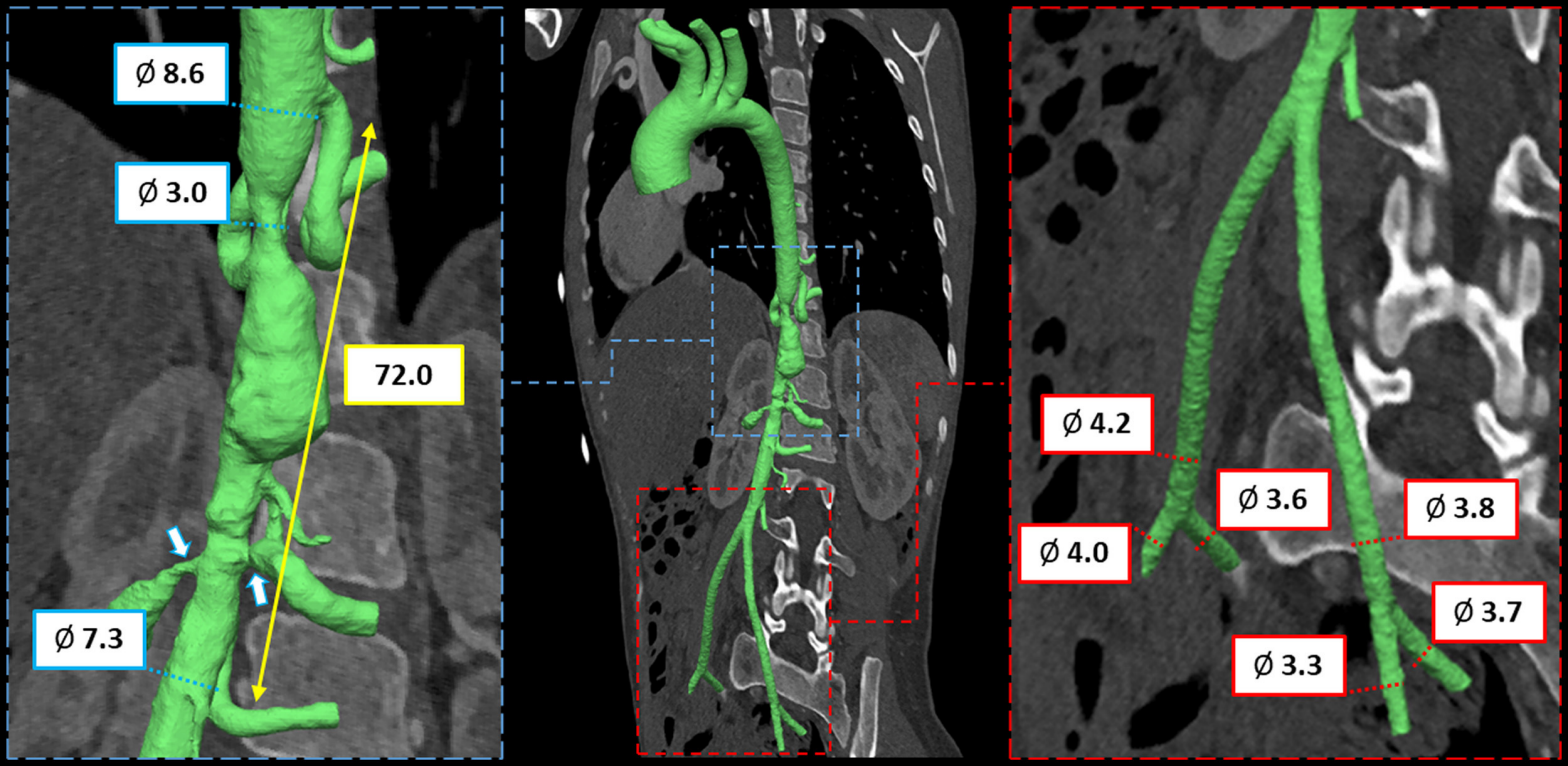
CT imaging and preoperative 3D anatomical model of mid-aortic clinical scenario with virtual measurements, expressed in mm.

To this purpose, CT scan derived preoperative anatomy was reconstructed (Mimics Medical, Materialise, Leuven, Belgium; Supplementary Video 1) and measures were extracted to assess size and length of the aortic segment to be treated and to evaluate the dimension of vascular access (Figure 1).

**Figure 2:**
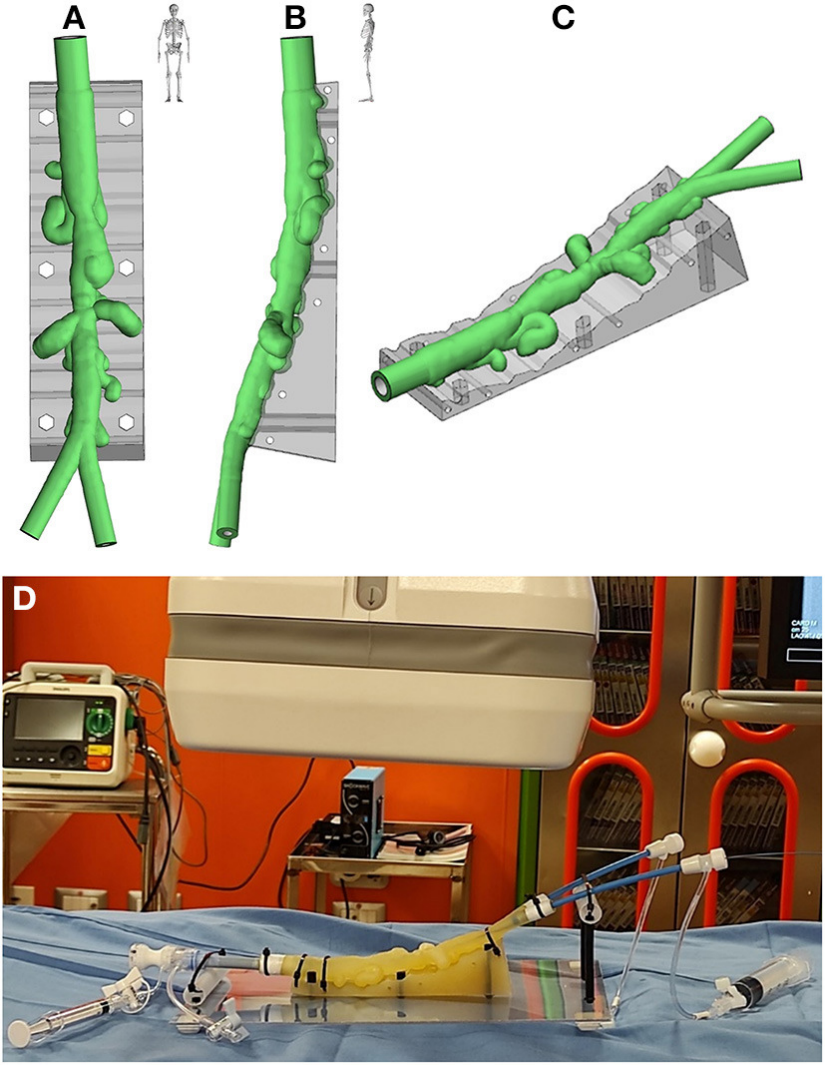
Antero-posterior **(A)**, latero-lateral **(B)**, and isometric **(C)** views of the virtual rendering of the 3D-printed model, replicated from patient-specific CT imaging; **(D)** 3D-printed model mounted on the *in vitro* planning platform.

To investigate the feasibility of a stenting procedure, an *in vitro* novel planning platform was designed (Figures 2A–C) and a translucent flexible 3D model was printed in-house (ProJet MJP2500, 3D Systems, Rock Hill, SC, USA; Supplementary Video 2). Specifically, we employed the VisiJet^®^ M2 ENT material, which is a soft rubber-like translucent elastomer, frequently employed for medical prototyping. The complete material datasheet with the full suite of physical, mechanical, thermal and electrical properties (as given per ASTM and ISO standards by the vendor), is available in the Supplementary Table 1. The model was placed on a 3D-printed support reproducing patient-specific clinostatis and a set of introducers were used to fill the model with saline and mimic fluid-catheter interaction (Figure 2D).

**Figure 3:**
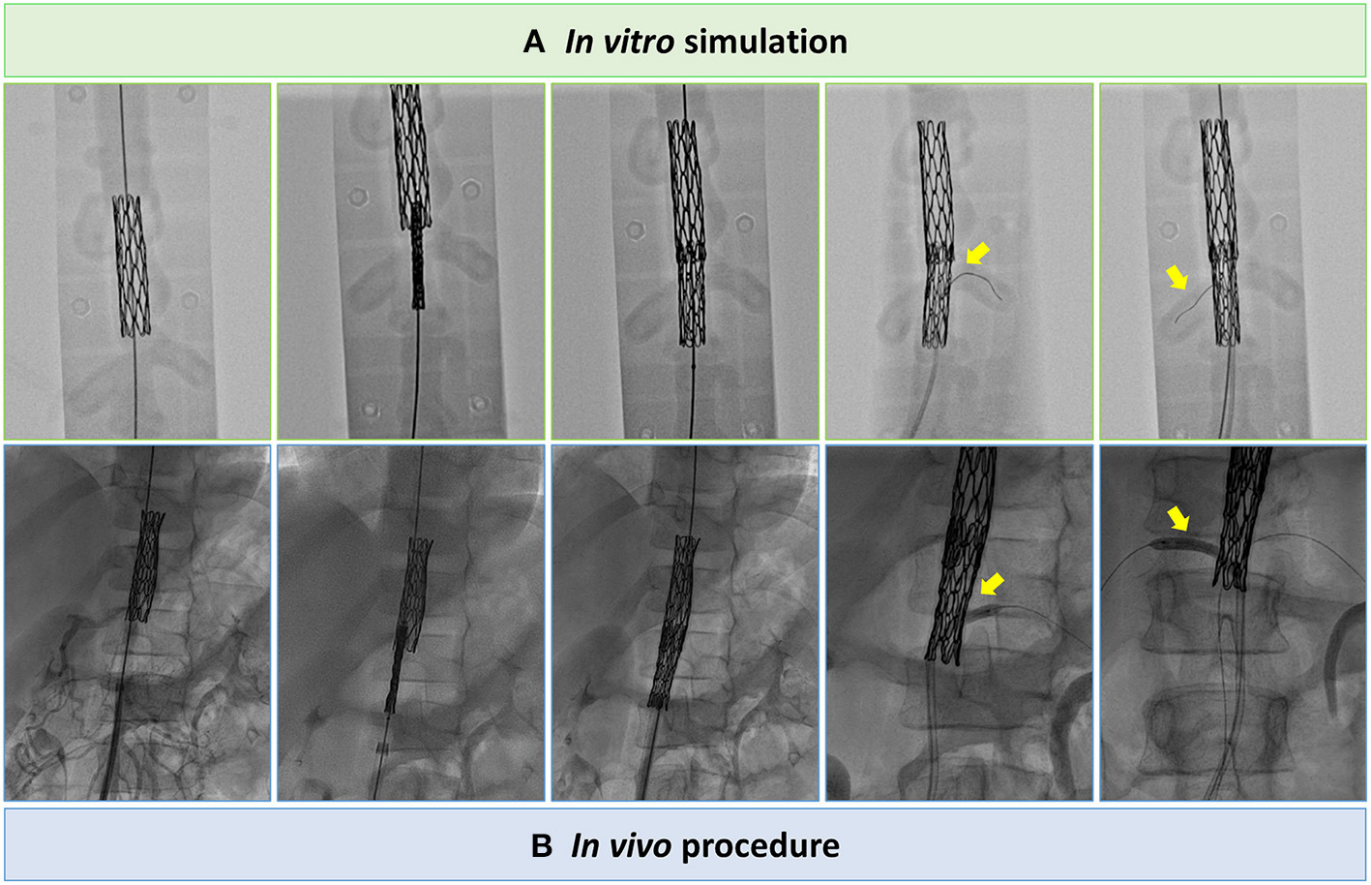
Still angiographic frames of the tests on the *in vitro* planning platform **(A)** to assess procedure feasibility and procedural steps performed *in vivo* **(B)**, i.e., CP stents implantation with partial overlap in pre- and infra-renal segments and bilateral renal angioplasty across the frames of the CP stent.

The planning platform was then positioned on the operating table to simulate the intervention under fluoroscopic guidance (Supplementary Video 3). Specifically, to treat the stenoses excluding the aneurysm and avoiding risk of renal arteries occlusion, 8-zig, 45-mm-long covered Cheatham-Platinum (CP) stent (NUMED, Hopkinton, New York, USA), mounted on 9 x 60 mm Armada balloon (Abbott, Chicago, IL, USA), and 8-zig, 34-mm-long CP stent, mounted on 8 x 60 Armada balloon, were implanted with partial overlap in pre- and infra-renal segments, respectively (Figure 3A; Supplementary Videos 4, 5). Bilateral renal patency was successfully tested across the stent frame with a 0.014” guidewire. Based on measurements from the 3D model, the size of the femoral sheath was reduced to 11 Fr, crimping each stent with a heart valve crimper. In addition, to improve procedural safety, surgical cut down of femoral artery was performed.

**Figure 4:**
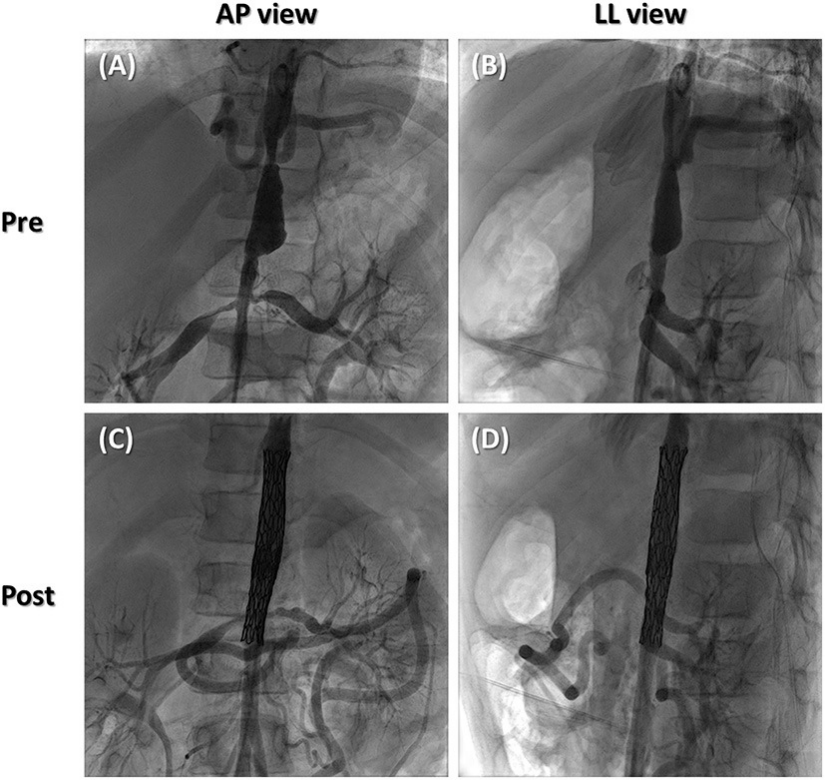
Still angiographic frames of preoperative scenario **(A, B)** and postoperative outcome **(C, D)** in antero-posterior **(A, C)** and latero-lateral views **(B, D)**.

During real cardiac catheterization (Figure 3B), the aortic stenoses were successfully treated (Figure 4), with no residual pressure gradient, exactly replicating the strategy tested *in vitro* (Supplementary Videos 6, 7). The cover stent was initially expanded to the nominal diameter of the 9 x 60 mm Armada balloon at 6 ATM in accordance with instructions for use of the manufacturer. This allowed to check aortic wall compliance while avoiding the risk of stent over-expansion with respect to the size of pre-stenotic aortic segment. Subsequently, post-dilatation was performed using the same 9 x 60 mm Armada balloon, inflated up to a maximum pressure of 12 ATM, so to minimize the risk of malposition between the stents' frames as well as the risk of intra-stent restenosis.

To improve bilateral renal flow, additional angioplasty was feasible across the frames of the stent (Supplementary Video 8) as investigated on the 3D-printed model. The frames of the uncovered stent were crossed by means of Hi-torque 0.014” balance middleweight (BMW; Abbott, Abbott Park, IL, United States) guidewires. Angioplasty of each renal artery was then performed using a coronary non-compliant Accuforce^TM^ 3 x 20 mm balloon (Terumo, Leuven, Belgium) inflated up to a pressure of 12 ATM. Postoperative course reported a systemic pressure reduction (130/70 mmHg) with a significantly reduced dosage of antihypertensive therapy.

## Discussion

Sen et al. (7) first described the clinical characteristics of MAS and designated this specific symptom-complex as “middle aortic syndrome.” When diagnosed in neonates, MAS is defined as a congenital disorder (8, 9), though MAS etiology can also be idiopathic or acquired, e.g., inflammatory disease, in young adults (6, 10). Suprarenal aortic narrowing is remarkably more frequent, i.e., up to 4 times, than infrarenal stenosis and more than 70% of pediatric patients diagnosed with MAS reported extra-aortic vessel involvement (4). Notably, as revealed by a systematic review of 630 reported cases of childhood MAS (1), aneurysm may also be present in MAS with an occurrence close to 11%. Furthermore, endovascular and surgical management of MAS are associated with a comparable mortality rate (in the range 2 ÷ 4%); conversely, the rate of complications in endovascular intervention can be threefold higher than abdominal open surgery, i.e., 27 vs. 9% of occurrence (1).

In this context, the case herein reported clearly represents a unique manifestation of the most critical features and challenges associated with MAS. Specifically, the young girl reported severe mid-aortic stenosis with a degree of obstruction equal to 59%, close to the threshold (mid-aortic stenosis ≥ 60%) reported by Porras et al. (4) for patients highly likely to require invasive management of MAS. Furthermore, the persistent severe hypertension reported by the patient was refractory to medical therapy, thus potentially exposing the patient to more serious complications such as stroke and congestive heart failure (11). Major concerns were also associated with the low weight of the baby (i.e., 27 Kg), which may increase the risk of vascular access damage, and the young age, already recognized as a risk factor for vascular complications (4).

Of note, our iatrogenic hypothesis about the origin of the aortic aneurysm mainly relies on the case-specific peculiarities. First, according to the clinical history of previous angioplasty procedures of the young patient, she was considered at high risk for the development of iatrogenic aortic aneurysm. In fact, aortic wall injury may frequently occur after angioplasty (12), also revealing an increased risk of aortic aneurysm in patients repaired at very young age and those who underwent multiple procedures (13). Second, as revealed by CT imaging, the single aneurysm with irregular aortic wall localized in correspondence of the site of previous interventions could corroborate the progression of acute aortic wall injury during follow up (14). Third, the incidence of aortic wall aneurysm in patients with idiopathic MAS (as the current case) is significantly lower with respect to MAS patients with arteritis, acquired inflammatory disease or genetic abnormalities (1). Nonetheless, a different origin of the aneurysm due to the natural progression of aortic pathology cannot be excluded.

The presence of aortic aneurysm between the suprarenal and infrarenal stenoses, with the latter also involving the renal arteries, represent the most critical issue for the planning of a percutaneous strategy of intervention. To this purpose, pre-procedural *in vitro* planning on the 3D-printed model offered valuable insight into the 3D spatial complexity of patient-specific anatomy and allow to define a percutaneous strategy able to exclude the aneurysm and preserve blood flow to the renal arteries. Indeed, taking pre- and post-stenotic dimension of thoraco-abdominal aorta into account, *in vitro* planning was crucial to test the feasibility of the stenting procedure, expanding each CP stent below the conventionally reported range of CP stent expansion. *In vitro* simulation demonstrated adequate lumen patency within both the expanded stents, no sleeve protrusion within the frames of the covered CP stent and sufficient unfolding of bare CP stent cells to avoid renal arteries obstruction. Of note, in the light of the verifications made, stent under-expansion with respect to vendor's recommendations was not considered a risk factor for restenosis because each stent was expanded in full accordance with the actual dimension of the vessel, i.e., the aortic pre- and post-stenotic segments, to achieve good apposition of the stents with the aortic wall. Also, to tackle the size of vascular access, stent crimping with a valve crimper proved to be effective, thus allowing to reduce the size of the femoral sheath to 11 Fr without causing damage to the stent.

Good qualitative agreement was noticed between the results of *in vitro* planning and the outcome of the *in vivo* procedure, thus corroborating both reliability and effectiveness of the proposed approach to plan a complex MAS intervention. Also, a personalized and reliable pre-procedural planning may provide additional advantages during the *in vivo* procedure: (i) procedure duration may be reduced thanks to a pre-designed and tested strategy, thus also reducing the time of anesthesia and potential associated risks; (ii) a lower dose of contrast agent may be enough to navigate *in vivo* the anatomy and perform the procedure, thus potentially reducing the risk of kidney injury as well as preserving end-organ function in MAS patients presenting with renal malperfusion.

Despite the satisfactory procedural outcome, freedom from reintervention may significantly reduce within the first 5 years of follow up (4). During follow up, reintervention could be due to uncontrolled systemic hypertension, re-stenosis of abdominal aorta or renal arteries, and intra-stent restenosis. Future dilatation of the implanted CP stents could be required, in accordance with the dimension of the aorta, due to the progressive growth of the young lady and the obvious lack of growth expected from stents. Also, additional stenting of the proximal renal arteries cannot be excluded. Conversely, surgical intervention, e.g., a bypass of renal arteries, would be unlikely due to the already recognized high surgical risk of the patient.

Despite the progressive diffusion of 3D-printed patient-specific models for preprocedural planning (15, 16), to the best of our knowledge, this is also the first report demonstrating the use of a 3D-printed model to plan percutaneous intervention in a complex pediatric MAS case, reliably reproducing the interventional scenario on a dedicated *in vitro* platform. This represents a technical improvement with respect to previous clinical case reports dealing with MAS (4, 6, 17–20), offering a novel and multi-disciplinary management strategy for MAS patients based on 3D technology.

It is worth noting that the 3D patient-specific anatomy was printed with an elastomeric material, which offers a certain level of vessel wall elasticity with respect to a conventional rigid 3D-printed model and allows to mimic the interaction of the stent with the vessel wall. Furthermore, the purposely designed 3D support and the filling of the 3D model with saline improved the realism of *in vitro* planning simulation, markedly enhancing clinical perception and device maneuverability as reported by the interventional cardiologists who practiced on the model. It is worth noting that, despite the not negligible cost of 3D-printed model production through in house facility (as in our case) or by means of outsourcing, all the auxiliary material and components required to assemble the *in vitro* planning platform were available in the catheterization laboratory. This allowed to perform the *in vitro* planning analysis with no need for more expensive experimental mock loop components (21).

Furthermore, the patient-specific 3D reconstruction and the tests performed *in vitro* also facilitated the communication with parents (given the very young age of the lady). They were informed about the complexity of preoperative clinical scenario using the 3D virtual reconstruction while the strategy of intervention was explained using the fluoroscopic images acquired during *in vitro* planning in the catheterization laboratory. Exploitation of virtual 3D-patient-specific models to facilitate communication in clinical practice is a clinically relevant area of research, which may enhance engagement with parents and improve their communication with cardiologists (22).

## Conclusion

The adopted *in vitro* platform, though less sophisticated than previous *in vitro* platforms for high-fidelity simulation (23), proved to be a valid solution to guide the planning of percutaneous intervention for MAS. Hence, personalized planning based on 3D-printed models and *in vitro* dedicated platforms is encouraged to test procedure feasibility and enhance intervention success.

## Data availability statement

The original contributions presented in the study are included in the article/Supplementary material, further inquiries can be directed to the corresponding author.

## Ethics statement

Ethical review and approval was not required for this case report study in accordance with the local legislation and institutional requirements.

## Author contributions

LG, GF, and FS contributed to conception and design of the study and wrote the first draft of the manuscript. GF, EV, and FS developed *in vitro* platform and elaborated 3D-printed patient-specific model. LG and MCa performed pre-procedural planning on the *in vitro* platform and data collection. MCh, EV, and MCa wrote sections of the manuscript and contributed to data interpretation and critical revision of the intellectual content. MCa and FS contributed to funding acquisition. All authors contributed to the revision of the final version of the manuscript, read, and approved the submitted version.

## Supplementary material

Supplementary Video 1 CT-derived preoperative reconstruction and assessment of patient-specific 3D aortic anatomy.

Supplementary Video 2 Virtual rendering of 3D patient-specific model of mid-aortic syndrome.

Supplementary Video 3 3D-printed model mounted on the *in vitro* planning platform for pre-procedural testing in the catheterization laboratory.

Supplementary Video 4 Covered CP-stent implantation during *in vitro* pre-procedural planning.

Supplementary Video 5 CP-stent implantation during *in vitro* simulation partially overlapping covered CP-stent; bilateral renal patency tested across the stent frames.

Supplementary Video 6 Implantation of covered CP-stent during *in vivo* procedure.

Supplementary Video 7 Implantation of CP-stent, partially overlapping covered CP-stent, during *in vivo* procedure.

Supplementary Video 8 Additional bilateral renal angioplasty across CP stent frames during catheterization procedure.

Supplementary Table 1 VisiJet^®^ M2 ENT material datasheet as officially provided by the vendor. VisiJet M2 ENT material datasheet available online (www.3dsystems.com, 3DS-50104A, 11-22).
