# Supplementary material for: Case report: Personalized transcatheter approach to mid-aortic syndrome by in vitro simulation on a 3-dimensional printed model
Source: Front Cardiovasc Med. 2023 Jan 10;9:1076359. doi: 10.3389/fcvm.2022.1076359 (PMC9871590; doi:10.3389/fcvm.2022.1076359)
Supplement: Supplementary Table 1 — VisiJet® M2 ENT material datasheet as officially provided by the vendor. VisiJet M2 ENT material datasheet available online (www.3dsystems.com, 3DS-50104A, 11-22). [file Table_1.docx]

**Supplementary materials**

**Supplementary Table S1**

VisiJet® M2 ENT material datasheet as officially provided by the vendor.

| **METRIC** | **ASTM** | | **ISO** | |
| --- | --- | --- | --- | --- |
| **PHYSICAL** | | | | |
| Solid Density | ASTM D792 | 1.12 g/cm³ | ISO 1183 | 1.12 g/cm³ |
| 24h Water Absorption | ASTM D570 | 0.9 % | ISO 62 | 0.9 % |
| **MECHANICAL** | | | | |
| Tensile Strength Ultimate | ASTM D638 Type IV | 1.4 MPa | ISO 527 -1/2 | 1.7 MPa |
| Tensile Strength at Yield | ASTM D638 Type IV | N/A | ISO 527 -1/2 | N/A |
| Tensile Modulus | ASTM D638 Type IV | 2 MPa | ISO 527 -1/2 | 9 MPa |
| Elongation at Break | ASTM D638 Type IV | >200% | ISO 527 -1/2 | >200% |
| Elongation at Yield | ASTM D638 Type IV | N/A | ISO 527 -1/2 | N/A |
| Tensile Stress (50% Elongation) | ASTM D638 Type IV | 0.16 MPa | ISO 527 -1/2 | N/A |
| Tensile Stress (100% Elongation) | ASTM D638 Type IV | 0.25 MPa | ISO 527 -1/2 | N/A |
| Tear Strength | ASTM D624 Type C | 4.7 kN/m | ISO 34-1 | 4.7 kN/m |
| Tear Strength | ASTM D624 Type T | 1.6 kN/m | ISO 34-1 | 1.6 kN/m |
| Shore Hardness | ASTM D2240 | 39 A | ISO 7619 | 39 A |
| Compression Set (%) 23C | ASTM D395 | 0.7 % | ISO 815-B | 0.7 % |
| Compression Set (%) 50C | ASTM D395 | N/A | ISO 815-B | N/A |
| Bayshore Rebound | ASTM D2632 | 8 % | - | - |
| **THERMAL** | | | | |
| Tg (DMA, E") | ASTM E1640 (E"Peak) | -5 °C | ISO 6721-1/11 (E" Peak) | -5 °C |
| CTE -50 to -15C | ASTM E831 | 85 ppm/°C | ISO 11359-2 | 85 ppm/K |
| CTE 0 to 50C | ASTM E832 | 206 ppm/°C | ISO 11359-2 | 206 ppm/K |
| **ELECTRICAL** | | | | |
| Dielectric Strength (kV/mm)  @ 3.0 mm thickness | ASTM D149 | 316 | - | - |
| Dielectric Constant @ 1 MHz | ASTM D150 | 4.46 | - | - |
| Dissipation Factor @ 1 MHz | ASTM D150 | 0.132 | - | - |
| Volume Resistivity (ohm-cm) | ASTM D257 | 1.54E+11 | - | - |
| VisiJet M2 ENT material datasheet available online (www.3dsystems.com, 3DS-50104A, 11-22). | | | | |
